# Supplementary figures and images for: Development of SNP Markers for Original Analysis and Germplasm Identification in Camellia sinensis
Source: Plants (Basel). 2022 Dec 29;12(1):162. doi: 10.3390/plants12010162 (PMC9824298; doi:10.3390/plants12010162)

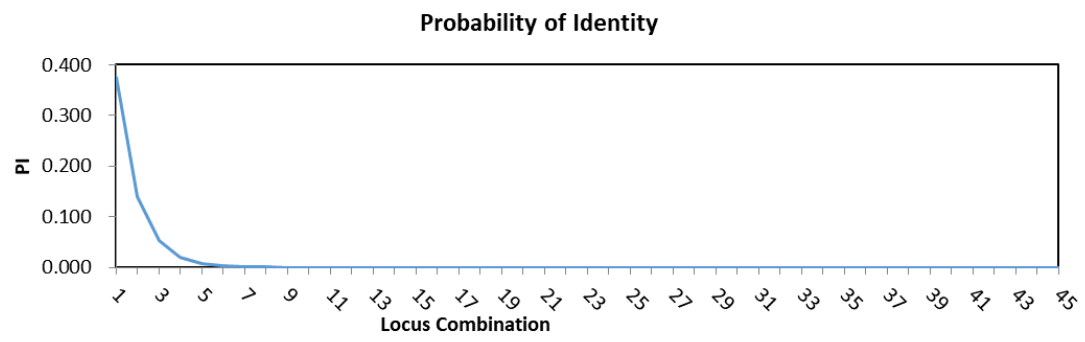

Figure S1: Probability of identity for increasing combinations of the 45 loci

Supplement: Supplementary file 1 [file plants-12-00162-s001.zip › Additional file/Figure S1.pdf]
